# What Is Known About the Nutritional Intake of Women during Pregnancy Following Bariatric Surgery? A Scoping Review

**DOI:** 10.3390/nu11092116

**Published:** 2019-09-05

**Authors:** Kate Maslin, Alison James, Anne Brown, Annick Bogaerts, Jill Shawe

**Affiliations:** 1 School of Nursing and Midwifery, University of Plymouth, Devon, PL4 8AA, UK; 2 Royal Cornwall Hospital Trust, Truro, Cornwall TR1 3LQ, UK; 3 Department Development and Regeneration, KU Leuven, Herestraat 49, 3000 Leuven, Belgium; 4 Faculty of Medicine and Health Sciences, Centre for Research and Innovation in Care (CRIC), University of Antwerp, 2000 Antwerp, Belgium

**Keywords:** metabolic surgery, bariatric surgery, pregnancy, maternal dietary intake, scoping review

## Abstract

Optimising the diet and weight of women prior to and during pregnancy is of paramount importance to both maternal and offspring health. In women who become pregnant after bariatric surgery, evidence suggests a better overall obstetric outcome in comparison to women with severe obesity managed conservatively. Historically, most studies in this population group have monitored supplement adherence or serum concentrations of micronutrients, rather than dietary intake. The aim of this study was to synthesise current knowledge of the dietary intake of women during pregnancy following bariatric surgery. A systematic search of search engines was conducted using the following databases: MEDLINE, Embase, CINAHL, Cochrane database, Scopus, Trip, NHS Evidence, UK Clinical Trials, ClinicalTrials.gov, Prospero, Epistemonikos and Open Grey. Titles and abstracts were screened independently by two reviewers against predefined inclusion and exclusion criteria. After removal of duplicates, 1594 titles were identified, of which 1586 were initially excluded. Following full-text review, four articles were included. In total, across all four studies, data from only 202 bariatric surgery participants were included, the majority of whom had had one type of surgery. Just one study included a control group. Reporting of nutritional outcomes was heterogenous, with none of the studies including complete macro and micronutrient intake results in their articles. An insufficient intake of protein was noted as a concern in two studies and associated with poor fetal growth in one study. Overall, this review has identified a paucity of data about the dietary intake of women during pregnancy after bariatric surgery.

## 1. Introduction

Optimising the diet and weight of women prior to and during pregnancy is of paramount importance to both maternal and offspring health [1]. Obesity in women has been linked to a number of adverse reproductive and birth outcomes [2,3,4]. The prevalence and severity of obesity in women of reproductive age is increasing in many regions of the world [5,6,7,8]. Bariatric surgery is a viable treatment option for people with severe obesity that can result in significant and sustained weight loss [9,10]. In the United States (US), the number of bariatric surgeries taking place has increased considerably from 2000–2012 [11], with 80% taking place in women between 1998–2010 [12]. A similar trend is evident in the United Kingdom (UK,) where the number of surgeries has increased thirty-fold since 2000 [13], with half occurring in women of childbearing age [14]. Research suggests that future pregnancy is important to 30.3% of women under 45 years awaiting bariatric surgery [15], suggesting that improving fertility may be a motivating factor for women seeking surgery. 

Nutritional intake post-bariatric surgery is dependent on a number of factors; including the type of surgery, symptoms (e.g., gastroesophageal reflux and pain [16,17]) and complications. Changes in taste preference [18], intolerance to specific foods [19] and persistence of disordered eating are also common [20]. There is a considerable risk of malnutrition if nutritional after care guidelines are not adhered to [21]. In women who conceive after surgery, pregnancy-related nausea and vomiting [22,23], combined with increased intra-abdominal pressure may worsen the risk of complications and poor nutritional intake [24,25]. Potential nutritional imbalances are complicated by the fact that preconception and pregnancy increase the demand for certain micronutrients such as folic acid [26], whilst other micronutrients need to be limited (e.g., retinol-based vitamin A) [27].

In women who become pregnant after bariatric surgery, evidence suggests a better overall obstetric outcome in comparison to morbidly obese women managed conservatively. This includes reduced risk of gestational diabetes and large-for-gestational-age infants; however, there is heightened risk of maternal nutritional deficiencies and infants born Small for Gestational Age (SGA) [28,29,30,31,32]. Historically, most studies in this population group have monitored women’s adherence to taking micronutrient supplements and/or serum concentrations of micronutrients, rather than dietary intake [33,34]. The purpose of this scoping review is, therefore, to provide a broad overview of research activity related to nutritional intake during pregnancy post-bariatric surgery, by systematically searching, selecting, and synthesising existing knowledge. We aim to determine what kind of evidence is available, map key concepts and identify gaps for future research. 

## 2. Materials and Methods

### 2.1. Study Design

This review used scoping methods, following the search strategy of the Joanna Briggs Institute [35] and the principles of Arksey and O’Malley’s framework [36].

The key phases of this approach are:

- identifying the research question
- identifying relevant studies
- study selection
- charting the data
- collating, summarising and reporting the results.

### 2.2. Identifying the Research Question

The scoping review addressing the following question: “What is known about the nutritional intake of women during pregnancy following bariatric surgery”?

### 2.3. Search Strategy

A systematic search of studies that measured dietary intake of pregnant women post bariatric surgery was carried out in November 2018. The search strategy was designed to find both published and unpublished studies in any language from 1980 to November 2018. The year 1980 represents the start of the decade in which the earliest publications about pregnancy following bariatric surgery appeared in the literature.

A keyword search strategy based on Population, Intervention and Outcome, combining terms associated with pregnancy, nutritional intake and bariatric surgery was created through an iterative process. An experienced medical librarian (author 3) was consulted and advised on the most appropriate Medical Subject Headings terms for the search and how to modify them for the different databases used. Based on this exploratory phase, the search strings for each database were finalised (Supplementary Material File S1).

A three-step search strategy was used, following the Joanna Briggs Institute approach [35]. The first step was an initial limited search of MEDLINE and CINAHL. This initial search was then followed by an analysis of the text words contained in the title and abstract of retrieved papers, and of the index terms used to describe the articles. A second search using all identified keywords and index terms was then undertaken across the following databases: MEDLINE (via Ovid), Embase (via OVID), CINAHL (via EBSCOhost, USA), Cochrane database (via Wiley, USA), Scopus, and TRIP. Additional searches were performed in NHS Evidence, UK Clinical Trials, ClinicalTrials.gov, Prospero and Epistemonikos databases. Grey literature was searched via Open Grey. Articles were retrieved from each database and imported into a reference management software.

Two independent reviewers (author 1 and author 2) screened the titles and abstracts of all papers identified to determine if they met the selection criteria. Both experimental and observational epidemiological study designs were included. No limits were placed on parity, body mass index, type of surgery or age of participants. Studies were included if they measured nutritional intake during any trimester of pregnancy, over any time period, using any method. Studies that were focused solely on intake of nutritional supplements or prevalence of micronutrient deficiency were excluded. Any disagreements were resolved through discussion.

Of the screened articles, full texts were retrieved, and these were assessed again by the same two independent reviewers. The reference lists of the included articles were hand-searched to detect any additional studies. Authors were contacted by email to clarify any queries regarding study populations. In the case of multiple publications on the same study population, the most recent paper or the paper describing the most complete dataset was included.

### 2.4. Charting and Summarising the Data

Data were extracted from the full-text papers by two independent reviewers (author 1 and author 2), using a predefined data collection proforma. The data collection form contained information on study characteristics (e.g., author, year of publication), design and methods (e.g., population characteristics, inclusion and exclusion criteria), exposure, outcome measures, results and conclusions/recommendations.

## 3. Results

**Figure 1:**
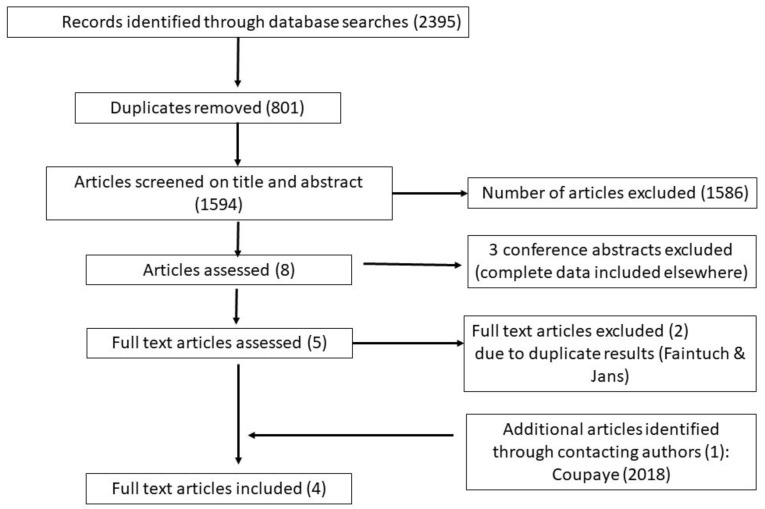
Preferred Reporting Items for Systematic Reviews and Meta-Analyses (PRISMA) flowchart.

The search strategy identified 2395 references, as shown in Figure 1—Preferred Reporting Items for Systematic Reviews and Meta-Analyses (PRISMA) flowchart. After removal of duplicates, 1594 unique references remained. Based on title and abstract screening, 1586 articles were excluded. Of the remaining eight articles, three were excluded as they were conference abstracts [37,38,39], of which a more complete dataset was reported in a subsequent included publication. Two articles [40,41] were excluded on the basis of full text, due to duplicate results reporting in another two retrieved articles [42,43]. One study was identified through author contact (identified by a current study listed on Clinical Trials.gov website) [44]. In total, four articles are included in this review.

The characteristics of the included articles are shown in Table 1. Table 2 charts the data extracted from the studies in more detail.

### 3.1. Study Characteristics

In total, the four included studies comprised of a total of 227 participants, of whom 202 had bariatric surgery. The majority of surgical participants (*n* = 145) had Roux en Y Gastric Banding (RYGB) surgery. Individual study samples ranged from 14 to 85 participants. One study was conducted in Brazil, two in Belgium and one in France. Two studies were prospective cohort studies [43,45] and two were retrospective case series reports [42,44]. Only one study had a control group [43].

### 3.2. Dietary Assessment Methodology of Included Studies

Three studies used food diaries, ranging between 3 and 7 days of recording [43,44,45]. The methodology was unclear in one study, with the authors stating: “energy and protein ingestion transcribed from patient’s chart” [42]. Three studies collected dietary information at more than one time point [42,43,45]. Guelinckx et al. [45] measured dietary quality using the Healthy Eating Index (HEI) and was the only study to report on food groups consumed.

### 3.3. Macronutrient and Micronutrient Intakes

None of the four studies reported a thorough breakdown of macronutrient and micronutrient intake. Dias et al. [42] reported only protein and energy intake. Similarly, Coupaye et al. [44] reported only energy and macronutrient intakes. The other two studies [43,45] reported macronutrient and selected micronutrient intakes (namely, calcium, iron, folate and vitamin B12). Two studies reported insufficient protein intake [42,44], with one study reporting an excessive percentage of energy that was derived from protein [45]. Both Jans et al. [43] and Guelinckx et al. [45] determined that saturated fat intake was excessive compared to national recommendations, with monounsaturated and polyunsaturated fats below recommended levels in the only study to report them [43]. Guelinckx et al. [45] assessed overall dietary quality, concluding that most participants required dietary improvements. Fibre and calcium were lower than recommended for pregnancy, although iron intake was sufficient. Intakes of folate, vitamin B12 were below national recommendations [43].

### 3.4. Group Comparisons

The only study to include a control group for comparison purposes [43], showed no difference in intake between groups in trimester 1. However there was a lower intake of polyunsaturated fat in the surgical group compared to the control group in trimester 3. Dietary quality did not differ between surgical types, with the exception of higher intakes of grains in the participants who had had Laparoscopic Adjustable Gastric Banding (LAGB) compared to the RYGB procedure. Coupaye [44] did not find any difference in energy, carbohydrate and fat intake according to surgical type.

### 3.5. Links between Nutritional Intake and Other Outcomes

One study investigated the association between dietary intake during pregnancy and fetal growth, concluding that fetal growth after bariatric surgery is positively associated with maternal protein supply and negatively correlated with maternal iron status [44]. One study investigated the association between dietary intake and pregnancy anxiety, finding no link with an inadequate maternal diet [41].

## 4. Discussion

This systematic search of literature identified only four studies with unique datasets that met the inclusion criteria, two of which were conducted by the same research group. Reporting of nutritional outcomes was heterogenous.None of the studies included complete macronutrient and micronutrient intake results in their articles. In total, across all four studies, data from only 202 bariatric surgery participants were included, the majority of whom had had one type of surgery (RYGB). Just one study included a control group. Studies took place in three countries, with no studies from a UK- or US-based population. Access to surgery, clinical protocols and nutritional guidelines differ between countries and regions of the world [24,46,47,48,49]; therefore, data from one country may not be generalisable to other populations.

It is recommended that pregnancy should be delayed for 12–18 months after surgery to reduce the potential for fetal malnutrition [21,24,47,50], although it is recognised that maternal age should be considered and individual advice should be applied [51]. As a result of improved fertility after bariatric surgery, the risk of an unintended pregnancy may increase [52]; meaning preconception and early pregnancy nutrition in this population may be overlooked. In the UK, clinical review under the care of a multidisciplinary team, including a specialist dietitian, is recommended to continue for at least two years post-surgery [21,48]. In the US, it is recommended that follow-up continues for a period of 12 months [47]. However, in conjunction with the recommendation that conception should ideally not occur for >12–18 months post-surgery, women who follow this advice and become pregnant at a later stage may no longer be under the care of their surgical team and be lost in the care pathway. Of note, in the four full-text articles assessed in this review, the average time interval between surgery and conception was >18 months. However, the variability was wide, with a minimum time gap of 2 months reported by Guelinckx et al. [45].

Pregnant women with a history of bariatric surgery are, therefore, a unique and potentially high-risk obstetric population requiring specific tailored advice [24]. It is recommended that they are given intensive dietetic support, preferably by dietitians with experience of managing the nutritional complications of bariatric surgery, and closely monitored for nutritional deficiencies with supplementation given as indicated by close monitoring [51,53]. Specifically, “monitoring of food intake” is advocated [25]. Previous systematic reviews of dietary changes undertaken by pregnant women in the general population of women who have not had bariatric surgery suggest that age, education and pregnancy intention may be factors that predict adherence to dietary improvements during pregnancy [54]. The limited research identified by this scoping review suggests dietary patterns can be improved substantially [45]; however, due to small sample sizes, it has not been possible to stratify results according to demographic characteristics. There is also an absence of data on whether food intolerance [19] and altered taste preferences post-surgery [18] affect pregnant women disproportionately and whether there is any interaction with pregnancy-related cravings [55]. Improved understanding and characterisation of this population group would help to target services towards the women in most need of dietary support.

Previous studies of pregnant women have emphasised adherence with nutritional supplementation advice and the time gap between surgery and conception [33], with a lack of attention paid to food intake and specifically, the role of protein intake. A sufficient intake of protein is required to preserve lean mass during rapid weight loss, especially during pregnancy, where there may be increased physiological need [24]. Two of the four identified studies in this review reported that protein intake of participants was insufficient. One of the studies [44] based this finding on a recommendation of minimal protein intake of 60 g/day [47]. The other study [42] reported a mean protein intake of between 61.7–62.8 g protein/day; therefore, it is not known on what criteria this finding was based. Recommendations for daily protein intake during pregnancy after bariatric surgery are not available and may depend on the type of surgery and time lapse from surgery [24]. The recommendation of Mechanick et al. [47] is based on expert opinion for the general population post-bariatric surgery and is not specific to pregnancy. Notably, Coupaye et al. (2018) has highlighted that protein intake may be associated with infants being born SGA, underlining the importance of further investigation of food and macronutrient intake in pregnancy, not merely assessment of micronutrient supplement intake.

Depleted maternal concentrations of vitamins A, B12, K, folate and iron post-bariatric surgery, have been reported in a systematic review of Jans et al. in 2015 [33]. Their review identified 29 case reports and eight cohort studies; however, the quality of reporting was deemed to be weak. A more recent systematic review identified 27 studies, comprising 2056 women with pregnancies after bariatric surgery. Deficiencies were reported in maternal concentrations of vitamins A, B1, B6, B12, C, D, K, iron, calcium, selenium, and phosphorous [34]. The studies identified in our review reported intakes of only four micronutrients (calcium and iron [45], folate and vitamin B12 [43]). There is limited data published on intakes of other nutrients known to be important during pregnancy, such as zinc [56], iodine [57] or omega 3 fatty acids [58]. Research is needed in order to better understand nutritional requirements during this phase (and indeed post-partum and during breastfeeding). This will allow associations to be drawn with clinical outcomes in the offspring and provide evidence-based dietary advice. Further investigation of food group intake and analysis of dietary patterns is also warranted, in order to emphasise and optimise nutritional intake from food as much as possible, especially considering that adherence to micronutrient supplementation is poor [59]. The AURORA (bAriatric sURgery in women Of Reproductive Age) study, a prospective cohort study in Belgium, is an example of such a study that can provide data [41,60]; however, other similar studies internationally are also needed.

Overall, this scoping review has identified a paucity of rigorous research around the topic of nutritional intake during pregnancy post-bariatric surgery. However, it is worth noting that studies of nutritional intake following bariatric surgery in the non-pregnant population are also limited, as are studies that monitor change in diet before and during pregnancy in the general population [54]. Capturing dietary intake accurately is inherently challenging and limitations, such as under-reporting are well described [61]. A systematic review of nutritional intake post-bariatric surgery conducted in 2011 identified 10 studies, none of which were deemed to be of strong quality [62]. Heterogenous methods were used, primarily paper-based food diaries or food frequency questionnaires. All but one study used a retrospective approach, potentially leading to errors of recall bias and difficulties in estimating portion sizes. A more recent systematic review of nutritional intake post-bariatric surgery identified 18 studies [63]; however, the review was limited to studies that measured energy and macronutrient intake in participants before and after surgery. Critical appraisal of studies was not conducted and again, most of the studies identified used a retrospective paper-based dietary assessment method. The development of web-based tools to record and analyse dietary data has considerably advanced in recent years [61], potentially reducing participant burden and allowing for greater accuracy in reporting of portion sizes. Use of a validated electronic method with accurate visual representation of food images is particularly important when portion sizes may be restricted, as is typically the case post-bariatric surgery.

### Strengths and Limitations of Methodology

A scoping review is a broad question to determine the extent, range and nature of research activity in a specific field. The methods used in this scoping review were rigorous and transparent, following a defined methodology [35,64], with systematic searches undertaken by an experienced medical librarian and screening undertaken by two independent reviewers. As the scoping study method seeks to identify all relevant literature regardless of study design, its reach is comprehensive. However, since quality assessment does not form part of the scoping methodology, it does not necessarily identify research gaps where the research itself is poor quality or determine whether particular studies provide robust findings [36,64].

## 5. Conclusions

The evidence underlying the importance of nutritional intake and being a healthy weight before and during pregnancy is substantial [1,3,4]. Whilst bariatric surgery can improve the health of women with obesity who become pregnant, it is also a cause of nutritional risk in women of childbearing age with potential impact on the offspring. Overall, the four studies identified suggest that dietary intakes do not meet nutritional requirements of pregnancy and therefore need improvement. In particular, suboptimal intakes of protein and omega 3 fatty acids [58], not previously highlighted by other reviews, may be of concern. Prospective research is needed to determine the optimal dietary management for preconception and pregnancy following bariatric surgery [11,24,33], and how it is related to important pregnancy and childbirth outcomes. The need for further research study is particularly timely given the advancement in dietary assessment technologies [61], the recent emphasis on improving preconception care planning [1] and the high rates of women of reproductive age seeking bariatric surgery [14].

## Figures and Tables

**Table 1:** Study characteristics.

Authors, Year	Study Population	Location	Participants (N)	Mean/Median Age * (Years)	Pre-Pregnancy BMI (kg/m^2^)	Time Interval Surgery to Conception (Months)	Method
Dias et al. (2009) [42]	Women who conceived between 0–5 years after having RYGB surgery	Brazil	14	31.8 ± 6.5	Not stated	24.2 ± 21.6	Retrospective medical note review
Guelinckx et al. (2012) [45]	Pregnant women with a history of bariatric surgery	Belgium	49: 18 LAGB 31 RYGB	31 (25–36) (LAGB group) 30 (18–38) (RYGB group)	31 (22–44) (LAGB group) 25 (22–39) (RYGB group)	44 (4–108) (LAGB group) 22 (2–96) (RYGB group)	Prospective study: 7 day food diary collected in first and second trimester (weeks 7–12 and week 20)
Jans et al. (2018) [43]	Pregnant women with a history of bariatric surgery	Belgium	54: 45 RYGB 2 SG 2 LAGB 2 Scoparino procedure 3 not specified	29.4 ± 4.3	28.1 ± 5.1	45.6 ± 29.9	Prospective study: 3 day food record measured in first and third trimester (15 weeks and 32 weeks)
Coupaye et al. [44] (2018)	Pregnant women who had bariatric surgery and at least 1 nutritional evaluation	France	85 with dietary data: 55 RYGB 30 SG	33.4 ± 4.7 (RYGB group) 31.1 ± 4.8 (SG group)	31.2 ± 5.0 (RYGB group) 31.6 ± 6.8 (SG group)	31 ± 22 (RYGB group) 24 ± 18 (SG group)	Prospective: 4 day food diary

Age is mean ± years or median (range in brackets). BMI = Body Mass Index. RYGB = Roux en Y Gastric Banding surgery, LAGB = Laparoscopic Adjustable Gastric Banding, SG = Sleeve Gastrectomy surgery. * median values with interquartile range in brackets.

**Table 2:** Key outcome measures and covariates.

Authors	Control Group	Method of Dietary Monitoring	Dietary Outcome Measures	Results	Conclusions/Recommendations
Dias et al. (2009) [42]	No	Unclear–“energy and protein ingestion transcribed from patient’s chart” (Trimester 2 and 3).	Energy intake Protein intake	Trimester 2 mean daily intake: Energy: 1789 ± 659 kcal Protein: 61.7 ± 19.8 g Trimester 3 mean daily intake: Energy: 1881 ± 835 kcal Protein: 62.8 ± 19.2 g	Energy, but not protein intake during pregnancy was appropriate. Long term nutritional monitoring should be a priority and dietary recommendations are in clear demand.
Guelinckx et al. (2012) [45]	No	7 day food diary (Trimester 1 and 2)	Energy intake Protein as % energy Total fat as % energy Saturated fat as % energy Carbohydrate as % energy Fibre Calcium Iron Diet quality (HEI score)	Trimester 1 mean daily intake LAGB participants: Energy: 1971 ± 430 kcal Protein as % energy: 15.1 ± 1.5 Total fat as % energy: 35.3 ± 4.7 Saturated fat as % energy: 13.0 ± 2.6 Carbohydrate as % energy: 49.8 ± 5.4 Fibre: 19.5 ± 5.4 g Calcium: 822 ± 273 mg Iron: 11 ± 2 mg RYGB participants: Energy: 1786 ± 283 kcal Protein as % energy: 15.8 ± 2.1 Total fat as % energy: 35.9 ± 3.3 Saturated fat as % energy: 14.2 ± 2.3 Carbohydrate as % energy: 48.5 ± 3.9 Fibre: 17.5 ± 5.3 g Calcium: 702 ± 228 mg Iron: 9 ± 2 mg Trimester 2 mean daily intake LAGB participants: Energy: 1978 ± 472 kcal Protein as % energy: 15.5 ± 2.2 Carbohydrate as % energy: 49.4 ± 4.4 Total fat as % energy: 14.2 ± 2.3 Saturated fat as % energy: 13.6 ± 2.4 Fibre: 17.7 ± 5.8 g Calcium: 806 ± 384 mg Iron: 10 ± 2 mg RYGB participants: Energy: 1895 ± 542 kcal Protein as % energy: 15.1 ± 2.4 Total fat as % energy: 35.1 ± 4.0 Saturated fat as % energy: 14.2 ± 1.9 Carbohydrate as % energy: 49.9 ± 4.4 Fibre: 18.2 ± 3.5 g Calcium: 764 ± 370 mg Iron: 10 ± 30 mg	Mean daily intake of fat and saturated fat and protein higher than recommended levels. Carbohydrate intake lower than recommended. Fibre and calcium lower than recommended. Iron intake sufficient. HEI score did not change during pregnancy and was comparable between groups. During the first trimester, 15% of participants had a healthy diet, 82% required improvement and 3% had a poor quality diet. In the second trimester, 10% had a healthy diet and 90% required improvement. Nutritional advice and lifestyle coaching recommended for this high-risk population.
Jans et al. (2018) [43]	Yes 25 pregnant women with obesity	3 day food record (Trimester 1 and 3)	Energy intake Total fat intake Saturated fat intake MUFA intake PUFA intake n3 fatty acids n6 fatty acids Folate Vitamin B12	Trimester 1 mean daily intake: Energy: 1452.46 ± 415.99 kcal Total fat: 56.21 ± 19.65 g Saturated fat: 23.39 ± 8.81 g MUFA: 17.73 ± 7.00 g PUFA: 8.80 ± 3.75 g n3 fatty acids: 0.78 (0.12–2.06) * g n6 fatty acids: 5.42 (1.38–15.25) * g Folate: 177.25 (42.01–419.62) * mcg Vitamin B12: 2.94 (0.59–8.54) * mcg Trimester 3 mean daily intake: Energy: 1514.47 ± 503.69 kcal Total fat: 56.8 ± 20.50 g Saturated fat: 24.25 ± 9.33 g MUFA: 17.86 ± 7.39 g PUFA: 8.35 (2.86–24.34) * g n3 fatty acids: 0.64 (0.12–2.28) * g n6 fatty acids: 5.18 (1.16–19.76) * g Folate: 171.75 ± 68.04 mcg Vitamin B12: 2.92 ±1.29 mcg	Both surgical and obese groups consumed a diet high in saturated fatty acids and low in unsaturated fatty acids. Intakes of n3 fatty acids, folate and vitamin B12 were below Belgian dietary recommendations. Pregnancy following bariatric surgery induces high levels of anxiety that are not associated with an inadequate maternal diet.
Coupaye (2018) [44]	No	4 day self-reported food diary (Trimester 2)	Energy intake Protein intake Carbohydrate intake Fat intake	Mean daily intake: RYGB participants: Energy intake: 1385 ± 400 kcal Protein intake: 59.1 ± 20.9 g Carbohydrate: 164 ± 49 g Fat: 54.8 ± 19.1 g SG participants: Energy: 1222 ± 425 kcal Protein: 46.7 ± 14.5 g Carbohydrate: 152 ± 59 g Fat: 47.3 ± 20.3 g	Energy, carbohydrate and fat intake did not differ between those who had RYGB and SG. Protein intake was significantly higher in those who had RYGB, but still below the 60 g/day recommendation. Fetal growth after bariatric surgery is positively associated with maternal protein supply and negatively correlated with maternal iron status.

Values are mean ± standard deviation unless stated. * median values with interquartile range in brackets. HEI: Healthy Eating Index MUFA: Monounsaturated fatty acids. PUFA: Polyunsaturated fatty acids RYGB: Roux en Y Gastroplasty SG: Sleeve Gastrectomy.

## Supplementary Materials

The following are available online at https://www.mdpi.com/2072-6643/11/9/2116/s1, File S1: Full search strategy.
