# Supplementary material for: What Is Known About the Nutritional Intake of Women during Pregnancy Following Bariatric Surgery? A Scoping Review
Source: Nutrients. 2019 Sep 5;11(9):2116. doi: 10.3390/nu11092116 (PMC6770652; doi:10.3390/nu11092116)
Supplement: Supplementary file 1 [file nutrients-11-02116-s001.pdf]

Supplementary file 1: Full search strategy
Embase

1	bariatric surgery.mp.
2	weight loss surgery.mp.
3	obesity surgery.mp.
4	(antiobesity surgery or anti-obesity surgery).mp.
5	(gastroplasty or gastrostomy or gastroenterostomy).mp.
6	(gastric bypass or RYGB or Roux-en-Y).mp.
7	(sleeve gastrectomy or gastric sleeve).mp.
8	(gastric band* or LAGB or lap band or lap-band).mp.
9	(stomach stapl* or gastric stapl*).mp.
10	(biliopancreatic diversion or biliopancreatic bypass or duodenal switch).mp.
11	jejunoileal bypass.mp.
12	stomach bypass.mp.
13	1 or 2 or 3 or 4 or 5 or 6 or 7 or 8 or 9 or 10 or 11 or 12
14	exp bariatric surgery/
15	obesity/su [Surgery]
16	morbid obesity/su [Surgery]
17	exp biliopancreatic bypass/
18	exp gastroenterostomy/
19	exp gastrectomy/
20	exp Roux-en-Y gastric bypass/
21	14 or 15 or 16 or 17 or 18 or 19 or 20
22	13 or 21
23	pregnan*.mp.
24	(mother or matern*).mp. [mp=title, abstract, heading word, drug trade name, original title, device manufacturer, drug manufacturer, device trade name, keyword, floating subheading word, candidate term word]
25	(antenatal or prenatal or perinatal).mp.

26	pregnancy/ or exp pregnancy in adolescence/ or exp pregnancy, high-risk/ or exp pregnancy, multiple/ or exp pregnancy, unplanned/
27	perinatal care/ or prenatal care/
28	23 or 24 or 25 or 26 or 27
29	(nutrient* intake or macronutrient* intake or micronutrient* intake).mp.
30	nutrition*.mp. [mp=title, abstract, heading word, drug trade name, original title, device manufacturer, drug manufacturer, device trade name, keyword, floating subheading word, candidate term word]
31	(food* or diet*).mp. [mp=title, abstract, heading word, drug trade name, original title, device manufacturer, drug manufacturer, device trade name, keyword, floating subheading word, candidate term word]
32	eat*.mp.
33	exp Food/
34	nutrition assessment/
35	exp Diet/
36	nutritional requirements/ or nutritional status/
37	exp dietary intake/
38	food intake/ or drinking/ or eating/ or energy consumption/ or portion size/ (167601)
39	maternal nutrition/
40	nutrition/
41	nutritional value/
42	29 or 30 or 31 or 32 or 33 or 34 or 35 or 36 or 37 or 38 or 39 or 40 or 41
43	22 and 28 and 42

Medline

ID	Search Terms
S40	S20 AND S38 AND S39
S39	S26 OR S27 OR S28 OR S29 OR S30 OR S31 OR S32 OR S33 OR S34 OR S35 OR S36 OR S37
S38	S21 OR S22 OR S23 OR S24 OR S25
S37	(MH "Nutritive Value+")
S36	(MH "Maternal Nutritional Physiological Phenomena+")
S35	(MH "Eating+") OR (MH "Serving Size") OR (MH "Portion Size")
S34	(MH "Nutritional Status")
S33	(MH "Nutritional Requirements+")
S32	(MH "Nutrition Assessment")
S31	(MH "Diet+")
S30	(MH "Food+")
S29	eat*
S28	food* OR diet*
S27	nutrition*
S26	nutrient* intake OR macronutrient* intake OR micronutrient* intake
S25	(MH "Perinatal Care") OR (MH "Prenatal Care")
S24	(MH "Pregnancy") OR (MH "Pregnancy, Unplanned") OR (MH "Pregnancy, Multiple") OR (MH "Pregnancy, HighRisk") OR (MH "Pregnancy in Adolescence")
S23	antenatal OR prenatal OR perinatal
S22	mother* OR matern*
S21	pregnan*
S20	S13 OR S19
S19	S14 OR S15 OR S16 OR S17 OR S18
S18	(MH "Gastrectomy")
S17	(MH "Gastroenterostomy+")
S16	(MH "Biliopancreatic Diversion")
S15	(MH "Obesity/SU") OR (MH "Obesity, Morbid/SU") OR (MH "Obesity, Abdominal/SU")
S14	(MH "Bariatric Surgery+")
S13	(S1 OR S2 OR S3 OR S4 OR S5 OR S6 OR S7 OR S8 OR S9 OR S10 OR S11 OR S12)
S12	stomach bypass OR gastric bypass
S11	jejunoileal bypass
S10	biliopancreatic diversion OR biliopancreatic bypass OR duodenal switch
S9	stomach stapl* OR gastric stapl*
S8	gastric band* OR LAGB OR lap band OR lapband
S7	sleeve gastrectomy OR gastric sleeve
S6	gastric bypass OR RYGB OR RouxenY
S5	gastroplasty OR gastrostomy OR gastroenterostomy
S4	antiobesity surgery OR antiobesity surgery
S3	obesity surgery
S2	weight loss surgery
S1	bariatric surgery

CINAHL

Search ID#	Search Terms
S39	S19 AND S25 AND S38
S38	S26 OR S27 OR S28 OR S29 OR S30 OR S31 OR S32 OR S33 OR S34 OR S35 OR S36 OR S37
S37	(MH "Diet+")
S36	(MH "Nutritive Value")
S35	(MH "Maternal Nutritional Physiology")
S34	(MH "Eating") OR (MH "Portion Size")
S33	(MH "Nutritional Status")
S32	(MH "Nutritional Requirements+")
S31	(MH "Nutritional Assessment")
S30	(MH "Food+")
S29	eat*
S28	food* OR diet*
S27	nutrition*
S26	nutrient* intake OR macronutrient* intake OR micronutrient* intake
S25	S20 OR S21 OR S22 OR S23 OR S24
S24	(MH "Perinatal Care") OR (MH "Prenatal Care")
S23	(MH "Pregnancy") OR (MH "Pregnancy, High Risk") OR (MH "Pregnancy, Prolonged") OR (MH "Pregnancy, Multiple") OR (MH "Pregnancy, Unplanned") OR (MH "Pregnancy, Unwanted") OR (MH "Pregnancy in Adolescence")
S22	antenatal OR prenatal OR perinatal
S21	mother* OR matern*
S20	pregnan*
S19	S13 OR S18
S18	S14 OR S15 OR S16 OR S17
S17	(MH "Gastrectomy")
S16	(MH "Gastroenterostomy+") OR (MH "Gastrostomy")
S15	(MH "Obesity/SU") OR (MH "Obesity, Morbid/SU")

S14	(MH "Bariatric Surgery") OR (MH "Gastric Bypass") OR (MH "Gastroplasty") OR (MH "Jejunoileal Bypass")
S13	(S1 OR S2 OR S3 OR S4 OR S5 OR S6 OR S7 OR S8 OR S9 OR S10 OR S11 OR S12)
S12	stomach bypass OR gastric bypass
S11	jejunoileal bypass
S10	biliopancreatic diversion OR biliopancreatic bypass OR duodenal switch
S9	stomach stapl* OR gastric stapl*
S8	gastric band* OR LAGB OR lap band OR lap-band
S7	sleeve gastrectomy OR gastric sleeve
S6	gatric bypass OR RYGB OR Roux-en-Y
S5	gastroplasty OR gastrostomy OR gastroenterostomy
S4	antiobesity surgery OR anti-obesity surgery
S3	obesity surgery
S2	weight loss surgery
S1	bariatric surgery

Cochrane

ID	Search
1	((bariatric OR obes* OR antiobesity or anti-obesity or "anti obesity" OR "weight loss") near/5 (surg*))
2	gastroplasty or gastrogastrostomy or gastroenterostomy
3	gastric bypass or RYGB or Roux-en-Y
4	sleeve gastrectomy or gastric sleeve
5	gastric band* or LAGB or lap band or lap-band
6	stomach stapl* or gastric stapl*
7	biliopancreatic diversion or biliopancreatic bypass or duodenal switch
8	jejunoileal bypass
9	Stomach bypass
10	{OR #1-#9}
11	MeSH descriptor: [Bariatric Surgery] explode all trees
12	MeSH descriptor: [Obesity] explode all trees and with qualifier(s): [surgery - SU]
13	MeSH descriptor: [Obesity, Morbid] explode all trees and with qualifier(s): [surgery - SU]
14	MeSH descriptor: [Biliopancreatic Diversion] explode all trees
15	MeSH descriptor: [Gastroenterostomy] explode all trees
16	MeSH descriptor: [Gastrectomy] explode all trees
17	MeSH descriptor: [Biliopancreatic Diversion] explode all trees
18	{OR #11-#17}
19	#10 OR #18
20	pregnan*
21	mother* OR maternal*
22	antenatal or prenatal or perinatal
23	[mh ^Pregnancy] OR [mh "Pregnancy, Unplanned"] OR [mh "Pregnancy, Multiple"] OR [mh "Pregnancy, HighRisk"] OR [mh "Pregnancy in Adolescence"]
24	[mh "perinatal care"] OR [mh "prenatal care"]
25	{OR #20-#23}
26	nutrient* intake OR macronutrient* intake OR micronutrient* intake
27	nutrition*
28	food* OR diet*
29	eat*
30	[mh food] OR [mh diet]
31	[mh "nutrition assessment"]
32	[mh "nutritional requirements"]
33	[mh "nutritional status"]
#4	[mh "Eating"] OR [mh "Serving Size"] OR [mh "Portion Size"]
#5	MeSH descriptor: [Maternal Nutritional Physiological Phenomena] explode all trees
36	MeSH descriptor: [Nutritive Value] explode all trees
37	{OR #26-#36}
38	#19 AND #25 AND #37

TRIP

((title:(pregnan* OR mother* OR matern* OR antenatal or prenatal or perinatal or pregnan*))(title:(stomach bypass OR gastric bypass OR jejunoileal bypass OR biliopancreatic diversion OR biliopancreatic bypass OR duodenal switch OR stomach stapl* OR gastric stapl* OR gastric band* OR LAGB OR lap band OR lapband OR sleeve gastrectomy OR gastric sleeve OR RYGB OR RouxenY OR gastroplasty OR gastrostomy OR gastroenterostomy OR antiobesity surgery OR obesity surgery OR weight loss surgery OR bariatric surgery))(title:(nutrit* OR food* OR diet* OR eat* OR intake))) OR ((abstract:(pregnan* OR mother* OR matern* OR antenatal or prenatal or perinatal or pregnan*))(abstract:(stomach bypass OR gastric bypass OR jejunoileal bypass OR biliopancreatic diversion OR biliopancreatic bypass OR duodenal switch OR stomach stapl* OR gastric stapl* OR gastric band* OR LAGB OR lap band OR lapband OR sleeve gastrectomy OR gastric sleeve OR RYGB OR RouxenY OR gastroplasty OR gastrostomy OR gastroenterostomy OR antiobesity surgery OR obesity surgery OR weight loss surgery OR bariatric surgery))(abstract:(nutrit* OR food* OR diet* OR eat* OR intake))

Scopus

(TITLE-ABS-KEY (pregnan* OR mother* OR matern* OR antenatal OR prenatal OR perinatal))

AND

(TITLE-ABS-KEY (nutrit* OR food* OR diet* OR eat* OR intake OR meal* OR nutrient*))

AND (TITLE-ABS-KEY ((stomach AND bypass) OR (gastric AND bypass) OR (jejunoileal AND bypass) OR (biliopancreatic AND diversion) OR (biliopancreatic AND bypass) OR (duodenal AND switch) OR (stomach AND stapl*) OR (gastric AND stapl*) OR (gastric AND band*) OR lagb OR (lap AND band) OR lapband OR (sleeve AND gastrectomy) OR (gastric AND sleeve) OR rygb OR (roux-en-y) OR gastroplasty OR gastrostomy OR gastroenterostomy OR (antiobesity AND surgery) OR (obesity AND surgery) OR (weight AND loss AND surgery) OR (bariatric AND surgery)))

Clinical Trials

**Synomyns**
**pregnancy**
Gestation
**mother**
Maternal
mothering
**maternal**
mother
mothering
**nutrition**
Nutritional Status
nutritional state
Dietary status
nutritional science
Nutritional Study
**nutritonal**
**food**
**diet**
Dietary
**bariatric surgery**
Bariatric Surgical Procedures
Metabolic Surgeries
Weight Loss Surgery
**surgery**
Surgical
invasive procedures
operations
operative procedures
operative therapy
Surgically

Synomyns
**bariatric**
**bariatric surgeries**
Bariatric Surgical Procedures
Metabolic Surgeries
Weight Loss Surgery
**surgeries**
invasive procedures
operations
operative procedures
operative therapy
Surgical
Surgically
**bariatric**

Open grey

Bariatric surgery
Obesity surgery
Weight loss surgery
Gastroplasty
Gastric bypass
Gastrectomy
Biliopancreatic diversion
Gastric band
Roux-en-Y
